# Functional magnetic resonance imaging of the ascending stages of the auditory system in dogs

**DOI:** 10.1186/1746-6148-9-210

**Published:** 2013-10-16

**Authors:** Jan-Peter Bach, Matthias Lüpke, Peter Dziallas, Patrick Wefstaedt, Stefan Uppenkamp, Hermann Seifert, Ingo Nolte

**Affiliations:** 1 Small Animal Clinic, University of Veterinary Medicine Hannover, Foundation, Germany; 2 Institute for General Radiology and Medical Physics, University of Veterinary Medicine Hannover, Foundation, Germany; 3 Medical Physics, Carl von Ossietzky University Oldenburg, Oldenburg, Germany

**Keywords:** fMRI, Dog, Auditory pathways, Anesthesia

## Abstract

**Background:**

Functional magnetic resonance imaging (fMRI) is a technique able to localize neural activity in the brain by detecting associated changes in blood flow. It is an essential tool for studying human functional neuroanatomy including the auditory system. There are only a few studies, however, using fMRI to study canine brain functions. In the current study ten anesthetized dogs were scanned during auditory stimulation. Two functional sequences, each in combination with a suitable stimulation paradigm, were used in each subject. Sequence 1 provided periods of silence during which acoustic stimuli could be presented unmasked by scanner noise (sparse temporal sampling) whereas in sequence 2 the scanner noise was present throughout the entire session (continuous imaging). The results obtained with the two different functional sequences were compared.

**Results:**

This study shows that with the proper experimental setup it is possible to detect neural activity in the auditory system of dogs. In contrast to human fMRI studies the strongest activity was found in the subcortical parts of the auditory pathways. Especially sequence 1 showed a high reliability in detecting activated voxels in brain regions associated with the auditory system.

**Conclusion:**

These results indicate that fMRI is applicable for studying the canine auditory system and could become an additional method for the clinical evaluation of the auditory function of dogs. Additionally, fMRI is an interesting technique for future studies concerned with canine functional neuroanatomy.

## Background

As dogs cannot communicate their perceptions verbally, it is impossible for the veterinary practitioner to precisely evaluate canine auditory function during the standard clinical examination. Particularly unilateral deafness is difficult to detect. Several methods have been proposed to gain information about canine patients’ ability to hear, the most commonly used in veterinary practice today being the brainstem auditory evoked response (BAER) [1,2].

Especially when neoplastic, traumatic or inflammatory lesions of the middle and inner ear or central nervous system are suspected to be the cause of hearing disorders, it is of diagnostic benefit to acquire anatomical images of the patients’ auditory system in addition to the functional information provided by BAER. Magnetic resonance imaging (MRI) is an excellent imaging modality to obtain anatomical information about the ear and central auditory system in dogs [3,4]. However, conventional MRI does not provide any functional information about the auditory system.

Functional magnetic resonance imaging (fMRI) is a technique able to localize neural activity in the brain including the auditory system by detecting associated changes in blood flow [5-7]. To do this, fMRI utilizes the blood oxygenation level-dependent (BOLD) effect. The BOLD effect relies on the principle that increased neural activity in a region of the brain is followed by increased metabolic activity and blood flow in this area. The resulting rise in oxygen supply exceeds the augmented demand for oxygen, leading to an increased ratio of oxygenated hemoglobin to deoxygenated hemoglobin. This increase in oxygen saturation results in a signal rise in the regions of neural activity in special MRI sequences [8,9]. Hence, the combination of structural MRI and fMRI provides the possibility to gain morphologic information on the auditory system along with functional information on patients’ ability to hear.

BOLD fMRI has been used to investigate the auditory system in human listeners and other primates [5-7,10] as well as in songbirds [11,12] in a variety of studies. In addition to this, the technique was used to examine the auditory function of cats [13] and rats [14]. In dogs, there are various fMRI studies concerned with examining the visual system [15-17]. In one particular study, hand signs denoting the presence or absence of food were used as stimuli [18]. In another study the neural responses to acupuncture were examined [19]. However, to the authors’ knowledge this is the first study utilizing BOLD fMRI to investigate canine auditory function.

In the present study, ten anesthetized beagles were examined via MRI. During scanning dogs were presented with acoustic stimuli to obtain fMRI data to answer the following questions: 1) Is it possible to detect a BOLD signal change following acoustic stimulation in the brain of the dog? 2) Is it possible to assign a BOLD signal change to specific regions along the canine auditory pathway? 3) Are there significant differences between the results obtained with a sequence using sparse temporal sampling [20] and a continuous imaging sequence? 4) Are the measured results reliable enough to use fMRI as a means for the clinical evaluation of the canine patient’s neural response to sound?

## Results

Data from eight experimental sessions were processed. In each session, functional data were obtained with two different sequences. Three areas known to develop a detectable BOLD response to auditory stimulation in humans [21-24] were defined as ROIs: the medial geniculate nucleus (MGN), the caudal colliculus (CC) and the temporal cortex (TC).

**Figure 1:**
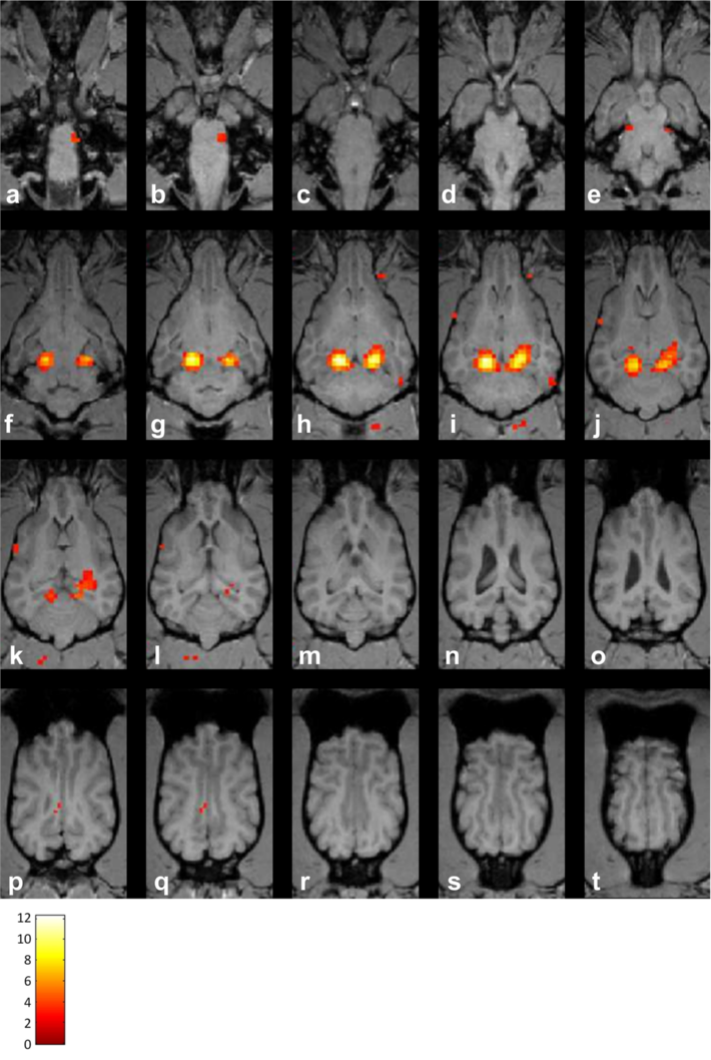
**Multiple dorsal sections from the brain of one dog with superimposed BOLD-activation following auditory stimulation (a-t).** T2* weighted functional data were obtained using sequence 1 to identify voxels responding to auditory stimulation. These voxels were afterwards superimposed on T1 weighted anatomical images of the subject (slice thickness between adjacent dorsal sections 2 mm) as colored pixels. The colorbar indicates the t-values of the activated voxels. The pictures show the most significant activation in the region of the caudal colliculi **(**slices **f**-**k)** followed by the medial geniculate nuclei **(**slices **h**-**k)**. Few activated voxels were found in the cortex and other regions of the brain. This activation pattern is representative for all functional data obtained with sequence 1.

**Figure 2:**
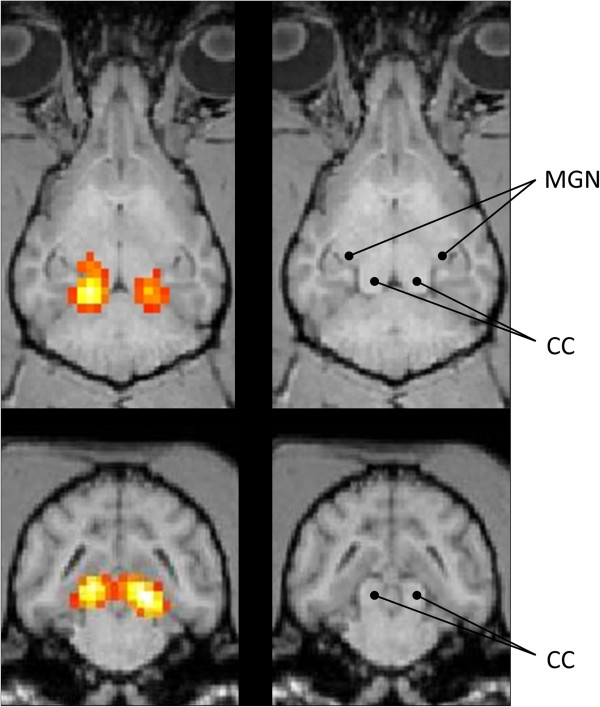
**Dorsal and transversal sections from the brain of another dog with superimposed BOLD-activation and labeling of anatomical structures.** The pictures show dorsal (upper row) and transversal (lower row) sections from the brain of one beagle. The position of the caudal colliculi (CC) and the medial geniculate nucleus (MGN) were identified using well-established anatomical literature [25,26]. The dorsal sections are taken at the level of the MGN and the lower CC and show activation in both CC and in the right MGN. The transversal images show bilateral activation of the CC. No cortical activation can be seen on the chosen sections.

Sequence 1 used the sparse temporal sampling method [20] to provide periods of silence in which the stimuli could be presented unmasked by scanner noise. With this sequence, significantly activated voxels could be found in all three regions of interest (ROIs) in all subjects. Concerning the activation found in the TC ROI it has to be noted that not all of the active voxels found in this ROI were located in areas commonly associated with an auditory function. The mean percentage signal change and the t-values calculated for the subcortical parts of the auditory pathways (CC and MGN ROI) were positive in all eight dogs, indicating a positive BOLD response of these areas following acoustic stimulation. In contrast to this, these values were negative in all but one subject for the TC ROI. Images obtained with this sequence are shown in Figures 1 and 2.

With sequence 2, in which no silent periods were included, significantly activated voxels were only evident in four dogs for the CC region, two dogs for the MGN region and five dogs for the TC region. Still, all but one beagle showed an increase in the percentage signal change and positive t-values for the subcortical ROIs and five for the TC region.

**Figure 3:**
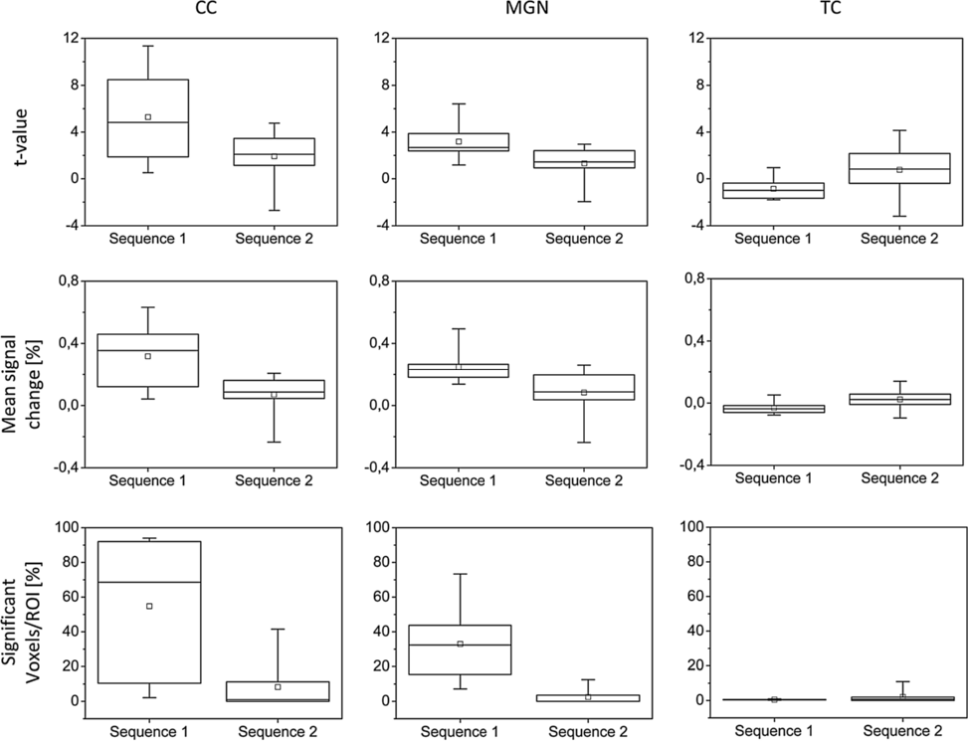
**Boxplots of data obtained during BOLD MRI with two different functional sequences.** Sequence 1 used the sparse temporal sampling method to provide periods of quiet for stimulus presentation by acquiring all images of a volume in rapid succession at the end of the stimulus and baseline conditions. In sequence 2 all volumes were recorded continuously. As a result the scanner noise was present throughout the whole scanning session. The caudal colliculi (CC), the medial geniculate nuclei (MGN) and the temporal cortex (TC) were chosen as Regions of Interest for further data analysis. T-values, the mean percentage signal change between active and passive state and the number of activated voxels at p-value 0.005 as a percentage of the total number of voxels within the ROI were calculated for each ROI. Bottom and top of the box display the 25% and 75% percentiles. The line inside the box marks the median, the ends of the whiskers the minimum and maximum of the data. The square represents the mean value.

Comparisons of t-values, mean percentage BOLD signal change and percentage of activated voxels/ROI between the two functional sequences used are shown in Figure 3. Sequence 1 showed significantly higher values in all three categories for the subcortical ROIs. There were no significant differences between the sequences for the TC ROI.

Apart from some activated voxels at the boundaries of the CC or MGN region, most patients showed little activation outside the ROIs, as expected.

## Discussion

BOLD fMRI provides a possibility to display brain regions responding to specific stimuli applied during the scanning session. It has become an essential tool for studying human auditory function [5-7,21-24] and has been successfully administered to other species like cats [13] and rats [14]. In this study, we applied this technique to anesthetized dogs.

The results of the study show that it is possible to elicit a detectable BOLD signal change in dogs with acoustic stimuli. This was possible despite several challenges involved with animal fMRI: One important aspect in animal fMRI is the requirement to prevent subject movement. Most studies in the past tried to do this by either immobilizing the animal [27,28] or using general anesthesia [13-17]. Recently, there was one study in which awake and unrestrained dogs were examined via MRI [18]. As it required a lot of training for the dogs to remain still in the scanner, this approach is not applicable in a clinical setting. Clearly, immobilization of an awake patient is not an option either: apart from possible neural activity induced by stress or other factors associated with lying in the MRI scanner it has to be rejected for ethical reasons, making anesthesia necessary for the clinical use of functional MRI in animals. Anesthesia depresses metabolic activity in the central nervous system and reduces the cerebral blood flow [29]. As the BOLD effect relies on an increased cerebral blood flow resulting in a change in the blood oxygenation level, a smaller signal change in the present study compared to studies in awake humans had to be expected. In a study in which the BOLD signal change in awake restrained rats was compared to that in rats anesthetized with propofol, a strong inhibition of the BOLD signal was observed with propofol [30]. Despite this, several studies succeeded in using functional MRI in anesthetized animals in the past [13-17,30]. These studies used different anesthetics to inhibit subject movement. Willis and Quinn used optical stimuli to compare different anesthetic regimens with regard to their suitability for fMRI studies in dogs [16]. These regimens used either injectable or inhalant agents for inducing and maintaining anesthesia. No significant differences were found between the anesthetic regimens, but five out of 36 experimental sessions had to be excluded due to subject movement. The current study used a combination of the injectable agents acepromazine, levomethadone and propofol for premedication and induction and the inhalant isoflurane for maintenance of anesthesia, combining the advantages of a fast induction with injectable anesthetics with the good controllability of inhalant anesthesia. With this anesthetic regimen it was possible to evoke a significant BOLD response in dogs. In addition to this, no data had to be excluded due to subject movement. This suggests that the combination of anesthetics used in this study is suitable for future fMRI studies in dogs.

The validity of the BOLD-activation elicited with the auditory stimulation paradigms in this study is supported by its spatial relation to specific regions along the auditory pathway: Most voxels showing a significant signal change following acoustic stimulation were found in brain regions which are known to be part of the auditory system. In the subcortical ROIs activated voxels were evident in all dogs using imaging sequence 1 and in seven out of eight dogs using imaging sequence 2. In contrast to the subcortical ROIs, the TC ROI, though considerably larger, only showed very few activated voxels in most subjects in this study. This is especially remarkable since in human auditory fMRI experiments the temporal cortex including primary auditory cortex and higher auditory areas is commonly the region showing the greatest signal change [22] and in many studies is the only region examined at all [5,7,20]. Stable activation of the auditory cortex following acoustic stimulation was also detected in cats [13] and rats [14]. The difficulties in detecting a signal change in the temporal cortex of the beagles in this study may have been caused by several aspects, the most important ones being anesthesia or possible interspecies physiological differences. In addition to this the studies detecting cortical activation in anesthetized cats and rats performed by Brown [13] and Cheung [14] used higher field strengths than the current study. As the auditory cortex eludes an examination with the BAER test, the improved representation of cortical activity evoked by auditory stimuli would be an additional advantage over traditional research methods.

Regarding the comparison of the two functional sequences used, sequence 1 provided significantly (p = 0.05) higher t-values, as well as a higher medium percentage signal change and number of significantly activated voxels for both subcortical ROIs than sequence 2 (Figure 3). This is in accordance with the results of previous studies, where sparse temporal sampling showed a higher BOLD signal change by up to 21% in comparison to continuous imaging methods at the cost of prolonging the acquisition time [31]. The superiority of the sparse temporal sampling method over continuous imaging is based on its ability to reduce the influence of the scanner noise on the acquired functional images: in a conventional MRI experiment with continuous acquisition of functional data, the intense background noise produced by the scanner is present throughout, resulting in a constant auditory stimulation of the subject even in the baseline condition. Due to this constant stimulation, the activation elicited by the auditory stimuli is harder to detect [32]. Though no significant differences between the two sequences were found for the TC region, the findings of this study indicate that sparse temporal sampling is suited for auditory fMRI studies in dogs.

Concerning the possible use of fMRI as a means for the clinical evaluation of the canine patients’ neural response to sound, it has to be noted that there were great variations in the level and spatial extent of the detected activation (Figure 3). As the clinical examination and the BAER test showed no hearing impairments in any dogs participating in this study, the cause for these variations is unclear. Though great effort was invested on adhering to a consistent experimental setup as closely as possible, several factors might have contributed to the differences in the level and spatial extent of the measured BOLD signals between the dogs: these include possible differences in the depth of anesthesia, the fit of the earplugs and ear covers, and the positioning of the surface coils. For a final assessment of the reliability and the possible clinical use of auditory fMRI in dogs, further studies including hearing impaired dogs and obtaining functional scans from the same subjects at multiple occasions are needed. In addition to this, a comparison of monaural and binaural stimulation and the investigation of the influence of different stimulus types and levels would be advantageous.

Apart from anesthesia, there are many aspects in canine auditory fMRI that might be optimized in future studies. These include the acoustic stimulation paradigm used, the choice and positioning of the MRI coils, the functional sequences, the measures taken to provide the best possible attenuation of scanner noise, and the postprocessing and data analysis procedures. For example, the hemodynamic response function used to model the expected signal course of a voxel responding to auditory stimuli was derived from human fMRI experiments. The development of a model function suited to dogs might improve the results of future canine fMRI studies. An advance that would surely be of great benefit would be the development of a standardized reference system for canine fMRI. In human fMRI studies, the collected data are commonly transferred to a standardized reference brain based on the Talairach coordinate system [33] and afterwards normalized. Normalization allows the combination of data across several subjects participating in a study, thus improving the study’s statistical power [8]. In addition to this, data acquired in different studies can easily be compared after normalization to the same reference system. Given the huge variation in size and shape of different canine species it seems unlikely that a single reference system can be developed which is suitable for all dogs. Due to the fact that the beagle is the dog breed most commonly used in animal studies [3,15-17,19], the development of a reference system representing the average anatomy of the beagle brain might be helpful for future studies. These optimizations and other future advancements in the experimental setup should allow for even better results when using fMRI in dogs and other non-primate animals.

In human medicine fMRI has proved to be valuable for the preoperative assessment of patients undergoing brain surgery and has become an essential tool for research in many clinical fields such as epilepsy and Alzheimer’s disease [34]. Auditory fMRI was the subject of clinical studies concerned with measuring the neurophysiological effects of tinnitus [23] and researching the functional adaptation to hearing loss [35]. In addition, it has successfully been used to estimate the potential benefit of cochlear implantation in hearing-impaired children [36]. Nevertheless, there have been few studies concerned with the use of fMRI in veterinary medicine. The results of this study demonstrate that useful fMRI data can be obtained in anesthetized dogs using auditory stimuli. This is encouraging for the future clinical and research use of canine auditory fMRI and fMRI in nonprimate animals in general.

## Conclusion

The results of this study indicate that functional MRI is suitable to detect a BOLD signal change in specific regions along the canine auditory pathway. Although fMRI cannot replace brainstem auditory evoked responses as an objective test of hearing, it has the potential to become an additional diagnostic tool for the clinical evaluation of the auditory function of dogs. Apart from that, fMRI is a powerful technique for future studies concerned with the canine auditory function and the functional neuroanatomy in general.

## Methods

The study was designed as a prospective, experimental study.

### Animals

Ten healthy male beagles (5 intact, 5 neutered) were included in the study with a mean age of 3.7 ± 2.3 years and an average body weight of 16.0 ± 2.6 kg. Prior to the fMRI experiments a general clinical examination and a neurological examination of the dogs were conducted. None of the beagles showed any neurological symptoms or signs of a reduced auditory sense or an increased anesthetic risk. Additionally, an otoscopic examination and an electrophysiologic audiometry using the BAER test (see below) were performed on each dog to ensure that the ear canals were not obstructed and the dogs were capable of hearing the stimuli. Two of the ten beagles were the subjects in a pilot study to test the experimental setup and optimize the scanning paradigm for the final study. All beagles included in the study were property of the University of Veterinary Medicine Hannover. All procedures were approved by the Animal Welfare Officer of the University of Veterinary Medicine Hannover and the Lower Saxony State Office for Consumer Protection and Food Safety, Oldenburg, Germany (TV-No. 33.9-42502-05-12A223).

### Anesthesia

For the fMRI experiments and the subsequent examinations the beagles were anesthetized using the following protocol: first, dogs were sedated using acepromazine (0.02 mg/kg i. m.) and a cephalic catheter was placed. Anesthesia was induced with levomethadone (0.2 mg/kg i. v.) and propofol (4–6 mg/kg i. v.). After inducing anesthesia, dogs were intubated and inhalant anesthesia was conducted (1% - 1.2% endtidal expired isoflurane). Intermittent positive pressure ventilation was performed with a tidal volume of 15 mL/kg, a respiratory frequency of 10 breaths per minute and a fresh gas flow of 150 mL/kg (equal parts of oxygen and room air).

To provide a stable and light depth of anesthesia, inspired and endtidal expired CO_2_ and isoflurane were recorded during all scanning sessions. In addition to this, a pulse oxymeter was used to monitor oxygen saturation and heart rate.

### BAER

For the BAER test, insert earphones were placed in both ears and hearing was assessed by delivering 100 μs monophasic click stimuli with alternating polarity at a rate of 11.1 Hz beginning at 90 dB sound pressure level (SPL) and decreasing in intensity in steps of 10 dB until the threshold was reached. Contra-lateral masking noise was delivered at 30 dB below the click stimulus level. The responses were amplified by a factor of 100,000 and band-pass filtered (150–3000 Hz). 1000 responses were averaged for each recording. The waves were labeled according to Steffen and Jaggy [37]. All dogs showed a detectable wave V at a stimulation level of 30 dB SPL and were therefore considered to have normal hearing.

### Stimuli

All sound stimuli were noise signals, which were bandpass-filtered between 250 and 4000 Hz, to provide broadband stimulation of the auditory system with no prominent spectral peaks. Two different types of noise were used. Half of the sounds were simply Gaussian noise stimuli, generated digitally using normally-distributed random numbers. The other half of the sounds were regular interval sounds, including a periodicity pitch as an additional sound feature. A regular interval sound (RIS) is created by delaying a copy of random noise and adding it back to the original in an iterative way. The resulting sound has some of the hiss of the original noise, but it also has a pitch corresponding to the inverse of the delay [38]. Gaussian noise and RIS are both known to create a stable BOLD response in human fMRI experiments [7]. A rest condition with no acoustic stimulus was also included to provide a baseline when identifying sound-related activation in the fMRI data.

To maximize the chance of eliciting a detectable BOLD response, all auditory stimuli were presented to the subjects binaurally with a level of 90 dB SPL via MR-compatible insert headphones (Sensimetrics S14 insert headphones, Sensimetrics corp., Malden). To provide additional hearing protection, the insert headphones were combined with canine ear covers (Mutt Muffs, Safe and Sound Pets, Westminster) with an estimated 25–28 dB sound reduction according to the manufacturer to protect the dogs’ hearing and to mitigate the effects of the scanner noise.

Two different activation paradigms were used, each in combination with a suitable fMRI sequence (see below).

Paradigm 1 included three different conditions: broadband noise, regular interval noise and silence, each condition lasting 10 seconds and repeated 40 times, giving a total of 120 trials. All sound conditions were presented in random order to exclude any unwanted effects of overall signal drift during the experiment, and to avoid possible systematic effects caused by the repeated presentation of identical stimuli [39].

**Figure 4:**
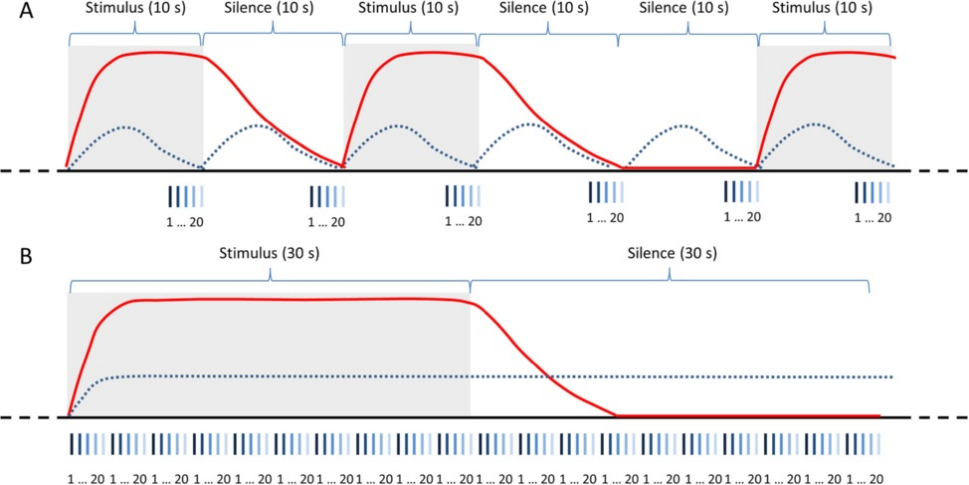
**Schematic comparison of the stimulus paradigms and sequences used.** A period of a minute of each functional sequence is shown. The acquisition of each functional volume (consisting of 20 slices) is illustrated by the small lines underneath the graphs. Cycles of auditory stimulation are denoted as gray shades while cycles without the application of auditory stimuli are shown in white. The red line represents the expected BOLD response to the stimuli. The expected response to the scanner noise is shown by the dotted lines. **(A)** Paradigm 1 involved ten-second cycles of stimulus and silence applied in random order. This paradigm was combined with sequence 1, which acquired all images of one volume in rapid succession at the end of each cycle, permitting the response evoked by the scanner noise to decay before the next image acquisition. This method of image acquisition is known as sparse temporal sampling [20]. **(B)** Paradigm 2 consisted of 30-second periods of stimulus and silence presented in alternating order. This paradigm was combined with sequence 2, which continuously generated scanner noise resulting in constant auditory stimulation even in periods in which no stimuli were presented.

Paradigm 2 consisted solely of regular interval noise and silence presented alternately. Each condition was repeated 8 times and lasted 30 seconds. A schematic comparison of the two stimulus paradigms along with their respective functional sequences is presented in Figure 4.

### Imaging

**Figure 5:**
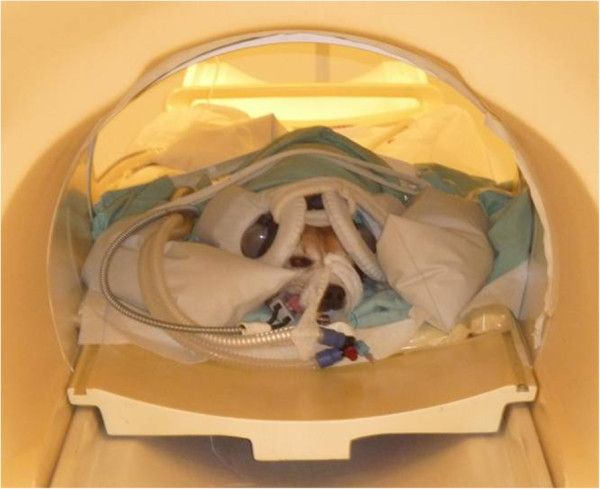
**Examination in the MRI scanner.** The dogs were placed in sternal recumbency in the MRI scanner and 11 cm diameter circular surface coils were placed laterally on each side of the dog’s head. Special canine ear covers were used to protect the dog’s hearing and reduce the effects of the background noise.

fMRI data were acquired on a 3 Tesla Philips Achieva MRI scanner in combination with 11 cm diameter circular surface coils (Figure 5). First, anatomical images of each dog’s brain were obtained using a T1 weighted sequence with repetition time (TR) = 11 ms and echo time (TE) = 5.2 ms with a field of view (FOV) of 220 mm and 0.7 mm × 0.7 mm × 0.7 mm isotropic voxels.

Directly following acquisition of anatomical data, images for functional evaluation were obtained using two different sequences in each dog (Figure 4). Both functional sequences were single shot echoplanar T2* weighted with a slice thickness of 2 mm for 20 contiguous slices in the dorsal plane, a FOV of 192 mm, a matrix of 96 × 96 and a flip angle of 90°.

Sequence 1: to reduce the effect of the scanner noise on the obtained images, the first sequence, which was combined with stimulus paradigm 1, used the sparse temporal sampling method [20]. During fMRI experiments, the scanner noise induces an auditory response in the subjects. Sparse temporal sampling utilizes the delay of the hemodynamic response to auditory stimulation to minimize the effects of the auditory response elicited by the scanner noise. To do this, sparse temporal sampling sequences contain gaps between image acquisitions, in which no scanner noise is present. During these gaps the different stimulus conditions are presented unmasked by scanner noise. Afterwards, all slices of a volume are acquired in rapid succession at the end of the stimulus and baseline conditions. Thus, the image acquisition is completed before the hemodynamic response to the scanner noise reaches a considerable level. Sequence 1 used a TE of 35 ms; a volume of images was obtained every 10 seconds (TR = 10 s), all images of one volume were recorded within a period of 3 seconds. One volume was collected for each of the 120 trials resulting in a duration of 20 minutes for this sequence.

Sequence 2: in the second sequence, which was combined with paradigm 2, all the slices of the volume were acquired at regular intervals with TR = 3000 ms and TE = 35 ms. With this sequence, 160 volumes were collected in 8 minutes.

### Data analysis

MRI data were processed and analyzed using SPM 8 [40]. Both functional data sets were processed separately. Prior to statistical analysis the functional volumes were spatially realigned to the first volume of the series and a mean functional volume was generated for each individual to which the anatomical images were then coregistered. The functional volumes were smoothed with a Gaussian Filter of 5 mm full width at half maximum. The anatomical volume was manually reoriented for presentation using a spatial transformation; afterwards the same spatial transformation was applied to the functional volumes.

The measured time course of the BOLD signal for each voxel was fitted using the general linear model, with the standard hemodynamic response function provided by SPM as a reference. All sound conditions combined were defined as active condition and contrasted with silence (no acoustic stimulus) as rest condition. The significance of the difference between conditions was then quantified by means of t-statistics, considering the mean and standard deviation of the estimated time course of the BOLD response for each condition.

An error probability of p < 0.005 (not corrected for multiple comparisons) was chosen as threshold for significance, which provided good results in a previous auditory fMRI study in anesthetized cats [13]. To avoid false positives and therefore further improve the validity of the results only clusters with at least three adjacent voxels that fulfill the criterion of a significant effect were interpreted as activated regions. The resulting clusters of activated voxels were then superimposed onto the anatomical images (Figures 1, 2).

After image processing was completed, three areas known to develop a detectable BOLD response to auditory stimulation in humans [21-24] were defined as ROIs: the medial geniculate nucleus (MGN), the caudal colliculus (CC) and the temporal cortex (TC). Due to the limited spatial resolution of the fMRI sequences used, no ROIs were created for smaller structures of the auditory pathways like the cochlear nuclei [34].

The region of the CC, which is easily identifiable on MR images, consisted of 2 bilateral cubic boxes with a side length of 6 mm. Palazzi’s 'The Beagle Brain in Stereotaxic Coordinates’ [25] and Assheuer’s 'MRI and CT Atlas of the dog’ [26] were used to identify the position of the medial geniculate nucleus and two cubic boxes with a side length of 4 mm were placed rostrally of the CC ROI as MGN region. The TC region was a larger rectangular volume with side lengths of 20 mm (dorsoventral and rostrocaudal) and 10 mm (mediolateral). This ROI was placed in the temporal lobe with the dorsal and caudal boundaries encompassing the dorsal and caudal extremities of the ectosylvian gyrus.

ROI analysis was performed using the SPM Toolbox Marsbar [41]. For each of the defined ROIs a mean value for all voxels in the ROI at each time point was calculated. The resulting time-course of the BOLD signal was then evaluated for contrasts between conditions using the general linear model. In addition to this, the mean percentage signal change between active and passive state and the number of activated voxels at p-value 0.005 as a percentage of the total number of voxels within the ROI were calculated for each ROI. A paired *t*-test (p = 0.05) was used to test differences in the results obtained with the two different sequences for significance.

## Competing interests

The authors declare that they have no competing interests.

## Authors’ contributions

ML, PW, HS and IN conceived and designed the study; JPB and PD performed the experiments; JPB, ML and SU analyzed the data; all authors read, contributed to and approved the final manuscript.
